# *Morus alba* L. for Blood Sugar Management: A Systematic Review and Meta-Analysis

**DOI:** 10.1155/2022/9282154

**Published:** 2022-05-23

**Authors:** Hye In Jeong, Soobin Jang, Kyeong Han Kim

**Affiliations:** ^1^Department of Preventive Medicine, College of Korean Medicine, Kyung Hee University, Seoul 02447, Republic of Korea; ^2^Department of Preventive Medicine, College of Korean Medicine, Daegu Haany University, Gyeongsan 38610, Republic of Korea; ^3^Department of Preventive Medicine, College of Korean Medicine, Woosuk University, Wanju 55338, Republic of Korea

## Abstract

**Introduction:**

*Morus alba* L. is used for blood sugar management in patients with diabetes mellitus. This review aimed to evaluate the effect of *Morus alba* on blood sugar management.

**Methods:**

This review was conducted in accordance with the Preferred Reporting Items for Systematic Review and Meta-Analysis Protocols (PRISMA-P). We searched PubMed, EMBASE, and four Korean medical databases (RISS, OASIS, NDSL, and KISS) using relevant keywords. Randomized controlled trials with any type of control intervention were included. The selection of studies, data extraction, and quality assessment were performed independently by two researchers.

**Results:**

Our results showed that *Morus alba* can reduce postprandial glucose and insulin levels. However, it is insufficient to conclude that *Morus alba* is an effective intervention for lowering blood glucose levels. Therefore, more rigorous studies are needed to reveal the effect of MA on blood glucose levels.

**Conclusion:**

The conclusion of this review provides evidence that *Morus alba* can control blood sugar level. This systematic review was registered with the International Prospective Register of Systematic Reviews (PROSPERO) (CRD42021255940).

## 1. Introduction

Diabetes mellitus (DM) is a major global health problem. International Diabetes Federation published the report about the expected rise of diabetic patients from 463 million in 2019 to 578 million by 2030 [1]. There are two types of DM: type 1 and type 2. Type 1 diabetes mellitus (T1DM) is characterized by deficient insulin production and requires daily administration of insulin. Type 2 diabetes mellitus (T2DM) results from the body's ineffective use of insulin. More than 95% of people with diabetes have T2DM [2].

The American Diabetes Association (ADA) recommends glycated hemoglobin A1C (HbA1C) below 7.0%, preprandial blood glucose concentration in the range from 80 to 130 mg/dL, and postprandial blood glucose level below 180 mg/dL for adults with diabetes [3]. Consistent hyperglycemia can cause diseases related to the heart, kidneys, eyes, and nerves; therefore, blood glucose should be managed well [4].

The Korea Diabetes Association updated clinical practice guidelines for Korean adults with DM. Patients with diabetes should receive education from multidisciplinary certified and professional education teams and constant monitoring for adherence to self-management, nutrition, and exercise. If blood glucose level is not controlled, the physician will need to prescribe oral antihyperglycemic agents or insulin. Sulfonylureas, metformin, glibenclamide, and thiazolidinediones are prescribed usually to patients with T2DM [5].

However, many adverse effects of these drugs have been reported. For example, glibenclamide can cause several episodes of hypoglycemia [6], and metformin is contraindicated in congestive heart failure because of the risk of lactic acidosis [7]. In particular, the use of common oral antidiabetic agents (ODAs) or oral hypoglycemic agents (OHA) in pregnant women remains controversial [8]. Therefore, diet and exercise are important for T2DM patients. Breakfast with adequate amounts of protein and fiber helps to maintain low blood glucose levels throughout the day [9]. Exercise has been shown to improve blood glucose control, reduce cardiovascular risk factors, contribute to weight loss, and improve well-being [10]. Herbal medicines could be effective in the management of carbohydrates in the normal diet and in reducing postprandial blood glucose levels [11].

The use of *Morus alba* Linn. (MA) was first recorded around 500 AD and has been used for more than 750 years in Japan as an infusion tea [12]. MA is used to reduce fever, treat sore throat, and improve eyesight [13, 14]. 1-Deoxynojirimycin (DNJ), a natural *α*-glucosidase inhibitor related to lower blood sugar, is most commonly found in mulberry leaves [15]. MA fruit extracts have neuroprotective, antioxidant, and antiobesity effects and prevent cardiovascular disease, immunomodulation, and antitumor activity [16–18].

This review aimed to evaluate whether MA can control blood glucose levels in humans. There is a systematic review on the effect of MA in improving blood glucose and lipid levels, but it includes compounds that combine with other substances [19]. Here, we evaluated the blood glucose meditating effects of MA alone.

## 2. Materials and Methods

### 2.1. Study Registration and Ethics Approval

The protocol for this systematic review was prepared according to the Preferred Reporting Items for Systematic Review and Meta-Analysis Protocols (PRISMA-P) guidelines and registered in PROSPERO under the number CRD42021255940. Data were collected from published studies; therefore, ethical approval was not required.

### 2.2. Search Strategy

The following databases were searched from inception to the current date: PubMed, EMBASE, and four Korean medical databases (RISS, OASIS, NDSL, and KISS). “*Morus alba*,” “Blood glucose,” and “Clinical trial” were the basic keywords for searching. In the Korean database, we used ((mulberry) OR (*Morus alba*)) AND (blood glucose) ((mulberry) OR (*Morus alba*) OR (mulberry extract)) AND (blood glucose (MeSH Terms)) AND (clinical trial) were applied to PubMed and EMBASE. We used MeSH terms in PubMed only.

### 2.3. Inclusion Criteria

#### 2.3.1. Types of Studies

Randomized controlled trials (RCTs) with any type of control intervention were included. Other designs, such as case reports, case series, non-RCT studies, animal and experimental studies, and reviews, were excluded. There were no restrictions on the year of publication.

#### 2.3.2. Type of Participants

Male or female participants of any age were included in the study. Individuals with impaired glucose tolerance (IGT) and T2DM were also included. Studies were excluded if the participants had other serious medical conditions, such as cancer, liver disease, and kidney disease.

### 2.4. Type of Interventions

Only the extract of MA, a white mulberry, was included. If the genera were the same and the species were different, such as black mulberry (*Morus nigra*), they were excluded. Any type of formulation (i.e., decoction, tablet, pill, or powder) of MA was eligible for inclusion. MA compounds with other herbal medicines or substances were excluded from this review.

#### 2.4.1. Type of Comparisons

There were no special restrictions on comparisons. All types of controls, such as placebo conventional treatment or no treatment, were included.

### 2.5. Outcome Measures

#### 2.5.1. Primary Outcomes

The primary outcomes are glucose-related figures:

1. The areas under the curve (AUC) after the oral glucose tolerance test (OGTT)
2. Blood glucose level, fasting blood sugar (FBS), hemoglobin A1C (HbA1C)
3. Insulin level

### 2.6. Secondary Outcomes

The secondary outcomes are Adverse events measured by any relevant incidence.

### 2.7. Data Extraction and Quality Assessment

#### 2.7.1. Selection of Studies

Two authors (HI and KH) independently screened the titles and abstracts of the searched studies after excluding duplicate articles. Then, the full text of the selected articles was reviewed to verify that each article met the inclusion criteria. If two authors had different opinions, the final decision was taken by another reviewer (SB).

#### 2.7.2. Data Extraction

Two authors extracted the following information: bibliographic information (e.g., author, publication date, and country), population demographics and setting (e.g., age, body mass index (BMI), and sample size), type of intervention (dosage, DNJ rate, type of sugar, and total duration), outcome measures, results, and adverse events. Disagreements were resolved by discussion among all authors, and SB acted as an arbiter.

#### 2.7.3. Risk Bias Assessment

The risk of bias was assessed using the Cochrane Handbook version 5.1.0, which considers random sequence generation, allocation concealment, blinding of participants and personnel, blinding of outcome assessment, incomplete outcome data, selective reporting, and other sources of bias. The results of the assessments were categorized as “high-risk (H),” “unclear (U),” or “low-risk (L).”

#### 2.7.4. Meta-Analysis

A meta-analysis was performed if sufficient studies were selected. We used the mean difference (MD) for continuous variables as effect estimates.

## 3. Results and Discussion

### 3.1. Results

**Figure 1:**
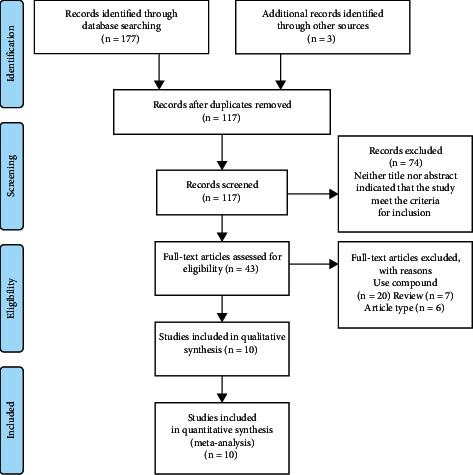
PRISMA diagram. From: Moher D, Liberati A, Tetzlaff J, Altman DG, The PRISMA Group (2009). Preferred reporting items for systematic reviews and meta-analyses: The PRISMA Statement. PLoS Med 6(6): e1000097. doi:10.1371/journal.pmed1000097

In total, 177 articles were identified from the six databases and previous meta-analysis reference list from which three articles [20–22] were retrieved. Of these, 117 articles remained after eliminating duplicates. After screening titles, abstracts, and reviewing the full text, 13 RCTs in 10 articles were eligible for inclusion. A flowchart of the study selection and exclusion criteria is shown in Figure 1. Three studies [23–25] explained two RCTs in one article at a time. Therefore, these three studies were regarded as six RCTs (Figure 1).

### 3.2. Included Studies

A detailed description of the characteristics of the included studies is given in Tables 1 and 2. The following is a brief overview of the studies. Data were extracted from the included studies.

### 3.3. Study Designs

Two study designs were included: short-term and long-term. A short-term study is a one-off design to test carbohydrate tolerance. These included 1–7 patient visits, with a gap for wash-out between visits. The gap ranged from 2 days to 2 weeks. A long-term study is a daily study for continuous days. The long-term study period was at least one month.

### 3.4. Settings

Of the 10 studies, three [20, 22, 26] were conducted in Korea, two each in United States [21, 27] and Japan [23, 25], and one each in UK [28], China [29], and Thailand [24].

### 3.5. Participants

The number of participants in the trials ranged from 10 to 85. There were three types of participants: healthy, individuals with IGT, and those with T2DM. Here, healthy means normoglycemic people, who do not have any type of DM or take any medicine, and have normal BMI and FPG. IGT refers to FBS levels from 100 mg/dL to 125 mg/dL. Two studies [23, 24] recruited individuals with FBS of 100–140 mg/dL. In 13 RCTs, five trials included healthy people, six included individuals with hyperglycemia, and one used both. Two trials reported that their target was T2DM. The average age of the healthy group was 20 years. If they had IGT or T2DM, the average age was 50 years. BMI varied according to the studies.

### 3.6. Interventions

The intervention used in all the studies was MA. The difference was in the formulation, which included powders, capsules, tablets, and just extracts. In five trials, capsule and powder were used most frequently. Extracts were used in two trials and tablets in one trial.

Long-term studies made participants take MA two or three times a day, and the dosage varied from 10.8 mg to 54 mg. In one trial [27], participants were advised to take 1 g MA 3 times a day before or during meals. In short-term studies, five studies had three treatment groups to test the MA dose difference. They tested doses ranging from 3 mg to 25 mg. Two other studies used 7.5 mg and 1 g, respectively.

Short-term studies used carbohydrates (CHO) to test glucose tolerance. The CHO types were glucose, maltose, sucrose, maltodextrin, and boiled white rice. While most studies used one type of CHO, especially sucrose, one study [29] used all of them. In seven short-term trials, four trials used 50 g CHO, two used 75 g CHO, and one used 200 g boiled white rice. The amount of water used as a solvent varied from 50 mL to 500 mL.

In short-term studies, participants in the four trials visited only once. In other trials, participants visited more than once, four times in two trials, and seven times in one trial. They had three different dose treatment groups. Therefore, for more accurate results, analyses in four trials were conducted with a wash-out period. However, the RCT by Thaipitakwong included only one test [24]; however, it had three dose groups. Several long-term studies had a run-in period and follow-up period.

### 3.7. Comparisons

Among 13 trials, 9 used a placebo as a control intervention. There were three placebos with no MA content, and other two used cellulose and red dye #40. Three trials did not report what was used as a placebo. No treatment was performed in four trials. However, in Thaipitakwong's long-term study [24], diet education was conducted for comparison with the treatment group.

### 3.8. Outcomes

The outcomes used to assess the effect of lowering blood sugar levels using MA included FBS, fasting plasma insulin (FPI), postprandial glucose (PPG), postprandial insulin (PPI), blood glucose level at 2 h postprandial (PP2hr), HbA1C, HOMA-insulin resistance (HOMA-IR), glycated albumin (GA), 1,5-anhydroglucitol (1.5AG), c-peptide, AUC, and glycemic excursion. Several studies used total cholesterol (T-chol), low-density lipoprotein (LDL), high-density lipoprotein (HDL), and triglyceride (TG) to assess lipid levels.

Long-term studies mainly used FBS, PPG, and HbA1C (four times in six trials). Four trials used FBS, and only one trial [28] reported that MA reduced FBS compared to the control group. However, it included diet education for the control group; thus, its influence cannot be ignored. The effect of MA on HbA1C was reported three times in four trials. The first was a comparison within the group. HbA1C measures the recent average blood glucose level; therefore, a decline in HbA1C implies that blood sugar has been well managed. The PPG was measured by various methods, including PP2hr, PPG from baseline to 120 min, self-monitoring, and glucose levels. In addition, PPI, c-peptide, LDL, incremental area under the curve (iAUC), and TG were reported by two studies [20, 26].

Seven short-term studies mostly used PPG and AUC. The AUC is an index of whole glucose excursion after glucose loading. It has been used to calculate glycemic index [30]. In seven studies, four used the AUC of glucose and five used PPG to examine blood sugar levels. Two studies [24, 29] used both the methods. AUC values were almost statistically significant, especially at high doses. Low-dose groups from 3 mg to 6.75 mg were not statistically significant.

PPG was similar to AUC because we used PPG to calculate AUC. Therefore, the results are similar. In four of the five trials, PPG was reduced at high doses. Three trials reported that insulin levels declined at high doses of MA. They used the positive incremental area under the curve (piAUC) and PPI. GI, glycemic excursion, and breath H_2_ concentration were reported by two studies [21, 29].

### 3.9. Adverse Events

Among the 13 trials, seven reported adverse events. Three short-term studies and four long-term studies reported various symptoms. Nausea, loose stool, constipation, proteinuria, and abdominal symptoms such as cramping, bloating, flatulence, and distension were reported.

### 3.10. Risk of Bias (RoB) in the Included Studies

**Figure 2:**
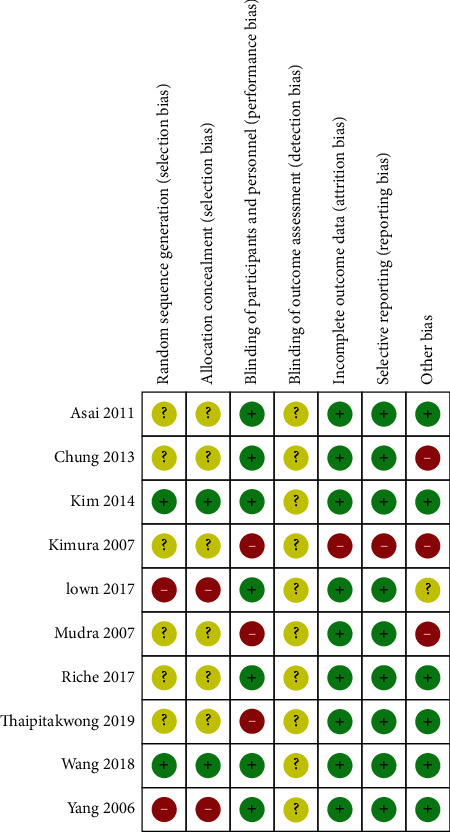
Risk of bias.

**Figure 3:**
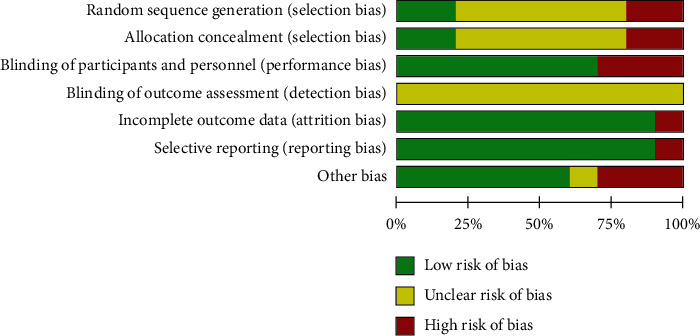
Risk of bias (graph).

The methodological quality of the included studies was assessed using the RoB tool, which evaluated seven areas. In random bias, only two studies [20, 29] explained the method used. Six studies [21–25, 27] did not report the exact methodology; thus, they were marked as “unclear.” The selection bias was similar to that with random bias. Seven studies used participant and assessor blinding and were assessed as “low-risk.” The detection bias was unclear because there were no pooled data. The majority of studies reported what they mentioned in the methodology section and thoroughly collected the outcome results. Thus, attrition bias and reporting bias were assessed as low risk. Additionally, other biases of four studies [21, 22, 25, 28] were assessed as high risk or unclear, since they did not adequately describe the baseline data. The details of the RoB assessment are shown in Figures 2 and 3.

### 3.11. Meta-Analysis

Due to the variety of outcomes, we included only seven studies [20, 23, 24, 26–29] in the quantitative synthesis. In short, MA was effective for iAUC of insulin. It also reduced PPG, but the heterogeneity was too high. MA did not improve FBS or HbA1C levels. The following is a brief overview of the meta-analysis results.

#### 3.11.1. iAUC of Glucose and Insulin

**Figure 4:**
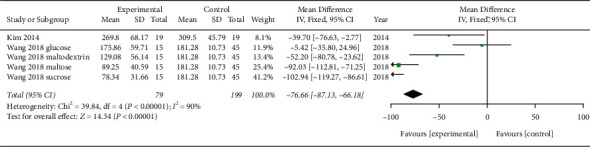
Meta-analysis (iAUC of glucose).

**Figure 5:**
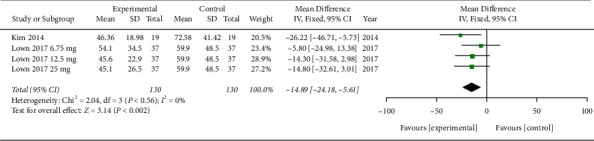
Meta-analysis (iAUC of insulin).

Five trials involving glucose and four involving insulin were suitable for the meta-analysis. Between the MA treatment and placebo groups, the mean differences in iAUC of glucose (MD −76.66; 95% confidence intervals (CI) −87.13, −66.18; *P*=0.00001; *I*^2^ = 90%; 278 participants; 5 trials) and insulin (MD −14.89; 95% CI −24.18, −5.61; *P*=0.002; *I*^2^ = 0%; 260 participants; 4 trials) were statistically significant (Figures 4 and 5).

#### 3.11.2. Fasting Blood Sugar

**Figure 6:**
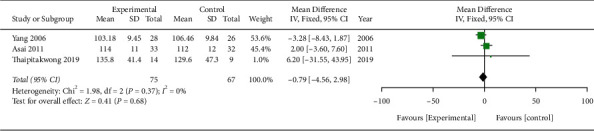
Meta-analysis (fasting blood sugar).

We compared the mean differences in fasting blood sugar levels between the MA treatment and placebo groups (MD −0.79; 95% CI −4.56, 2.98; *P*=0.68; *I*^2^ = 0%; 142 participants; 3 trials), and they were not statistically significant (Figure 6).

#### 3.11.3. HbA1C

**Figure 7:**
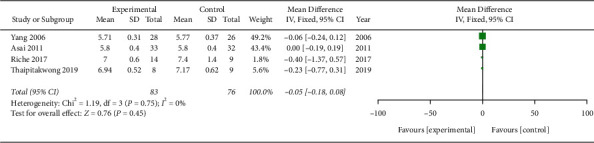
Meta-analysis (HbA1C).

Four trials included data on HbA1C (MD −0.05; 95% CI −0.18, 0.08; *P*=0.45; *I*^2^ = 0%; 159 participants; 4 trials); however, the results were not statistically significant (Figure 7).

## 4. Discussion

This study aimed to update the treatment effect of MA on DM by collecting data from clinical studies. In this review, we searched several databases to identify comprehensive data sources. The purpose of this review was to provide an overview of previous studies and investigate the effectiveness of MA. Thirteen trials from 10 studies were included in this review, and seven studies were suitable for meta-analysis. They have been conducted in Korea, the United States, Japan, the UK, China, and Thailand.

The studies were divided into two types. First was a short-term study, which is a one-off design to test carbohydrate tolerance. The other was a long-term study, which evaluated the daily intake effect of MA.

At first, we tried to include only healthy people. However, the definition of healthy varied among studies. For example, two studies [28, 29] defined healthy by FBS and Hb1AC levels, while another study [25] defined without using any specific criteria. Other studies [20, 22] included people with IGT by using the standard of FBS ≤125 mg/dL; therefore, the inclusion criteria among studies were ambiguous. Two studies [26, 27] included T2DM patients, but they chose people who had well-managed HbA1C levels between 7% and 8% or did not have any complications. Therefore, we did not focus on the physical condition but the exclusive use of MA.

There were no differences in the usage of formulations depending on the duration of the experiment. Each author would have used an easy-to-get or comfortable formulation.

In most studies, participants took MA in milligrams. Only two studies [21, 27] administered doses more than 1 g. One of them [21] clearly recorded MA doses, but another study [27] did not. The intake time varied in all studies, before, during, and after meals.

Regarding capacity selection, the studies used 50 g and 75 g of CHO. The 50 g dose may have been derived from a previous article about glucose examination [31]. In the PP2hr test, CHO was fixed at 75 g. So, all trials used either 50 g or 75 g of CHO.

Most of the side effects were related to the gastrointestinal tract. It seems to relate with characteristics of the experiments. Flatulence, bloating, and loose stool were the main symptoms. However, there were no serious adverse events, and the side effects gradually disappeared over time.

Blood sugar levels were measured intensively, but insulin and other indicators were also used. In particular, because of the relationship between sugar and lipids, there were some studies using the lipid levels [32].

The OGTT can demonstrate postchallenge glucose excursion, so it is highly sensitive and specific for detecting glucose intolerance. However, the PP2hr levels, a criterion for glucose intolerance in the OGTT, may not provide complete information regarding the processing of PP2hr after glucose loading. Whole glucose excursion is considered to provide more information about glucose tolerance rather than the PPG levels at a point [33].

The AUC is derived from the OGTT, which is widely used to diagnose IGT in clinical settings. According to the variations in fasting plasma glucose between individuals, the application of iAUC has been developed. However, iAUC obtained by subtracting the baseline value of fasting plasma glucose has been challenging because of the formation of negative values [34]. Therefore, piAUC has been suggested, and only values above the baseline value have been considered for application in the studies [35, 36]. Each method has its own strengths; therefore, they are all used evenly.

In short-term studies, it was difficult to achieve a blood sugar drop effect. Most studies showed statistically significant differences from the controls at 30 min. Wang's study [29] used four types of CHO, of which sucrose was the most effective for evaluating blood glucose. In addition, three other studies [21, 24, 25] used sucrose as CHO due to various physiological mechanisms. DNJ prevents hydrolysis and absorption of sucrose through inhibition of *α*-glucosidase activity, but not *β*-fructofuranosidase activity [15]. This might explain why the percentage reduction in the gastrointestinal tract was lower for sucrose than for maltose. In addition, maltodextrin has a multistep process, which may explain why the peak PPG level was achieved more slowly for maltodextrin than for maltose or sucrose [37]. Long-term studies showed higher improvement in HbA1C than in FBS or PPG. This can be seen as a reflection of the characteristics of long-term research.

As a result of meta-analysis, MA was not effective in controlling FBS and HbA1C but improved iAUC of insulin and PPG. We included seven studies in the quantitative synthesis for the variety of outcomes. Experiments with different capacities within one study were regarded as individual studies.

The following are strengths of this study. We analyzed the previous research by dividing it into long-term and short-term studies, so that the differences in experimental methods could be clearly established. We have not only summarized the results but also performed a meta-analysis. We modified both the unclear and ambiguous outcome values and representations of previous studies.

This review has several limitations. First, we collected data on individuals with IGT and T2DM. Although we limited the interventions to the sole use of MA, ununified participants can affect the combination of outcomes. Second, there are several kinds of bias. Last, there may be literature that was not retrieved because of the limitation of the search strategy.

## 5. Conclusions

According to this article, MA can reduce postprandial glucose and insulin levels. However, it is insufficient to conclude that MA is an effective intervention for lowering blood glucose levels. Therefore, more rigorous studies are needed to reveal the effect of MA on blood glucose levels.

## Figures and Tables

**Table 1:** Long-term studies.

Study ID	Characteristics of study				Interventions				Outcome	Result	Adverse events
	Sample size (I/C)	Subject health	Age (mean, SD)	BMI (kg/m^2^)	Period	Method	Intake	Control intervention			
Thaipitakwong (2019)	54 (28/26)	FBS 100–140 or PP2hr 140–199	t/53.14 ± 5.48 c/52.00 ± 8.22	t/30.06 ± 4.06 c/31.61 ± 5.85	12w + 4w (f/u)	Diet education + powder	4.6 g (12 mg) tid a.c	Diet education + N.T	FBS PP2hr HbA1C FPI HOMA-IR	+ − + − −	Bloating Flatulence Loose stool Constipation
Kimura (2007)	12 (6/6)	Healthy	24.7 ± 1.0	21.3 ± 0.6	38 days	Powder	1.2 g (18 mg) tid a.c	Placebo	Glucose level T-chol	− −	N.R
Asai (2011)	65 (33/32)	FBS 110–140	t/53.6 ± 5.8 c/53.5 ± 7.5	N.R	12w + 4w (f/u)	Capsule	6 mg tid a.c	N.T	FBS Insulin level HbA1C GA 1.5AG	− − +^∗∗^ +^∗∗^ +	Abdominal distension Modest proteinuria
Kim (2014)	38 (19/19)	FBS 100–125	t/53.0 ± 7.20 c/50.16 ± 7.83	t/24.69 ± 2.19 c/25.93 ± 3.86	4w	Tablet	3.6 mg tid	Placebo	FBS PPG PPI Glucose (iAUC) Insulin (iAUC) C-peptide (iAUC) Other blood parameters	− − + − + − −	N.R
Yang (2006)	23 (14/9)	T2DM	t/58.9 ± 8.7 c/61.8 ± 10.8	t/23.4 ± 3.2 c/25.5 ± 5.3	12w	Capsule	0.5 g (5 mg) bid	Cellulose	FBS HbA1C T-chol LDL HDL TG	− + − + − +	Indigestion
Riche (2017)	17 (8/9)	T2DM^†^	N.R	N.R	12w + 12w (f/u)	Capsule	1g tid	Placebo	SBP DBP HbA1C Weight SMBG	− − − − +^‡^	Stomach upset Influenza

GA, glycated albumin; 1.5AG, 1,5-anhydroglucitol; SMBG, self-monitoring blood glucose; SBP, systolic blood pressure; DBP, diastolic blood pressure; HOMA-IR, homeostatic model assessment of insulin resistance; PP2hr, blood glucose level at postprandial 2 hours; HDL, high-density lipoprotein; LDL, low-density lipoprotein ^*∗*^We use “+” if results were statistically significant and “–” if results were not statistically significant or have no difference. ^∗∗^Start to end, each group. ^†^Fairly controlled, HbA1C 7–8%. ^‡^MLE group versus baseline.

**Table 2:** Short-term studies.

Study ID	Characteristics of the study				Interventions					Outcomes	Result^*∗*^	Adverse events
	Sample size	Subject health	Age (mean, SD)	BMI (kg/m^2^)	Visit (times)	Method	Glucose (water)	Intake	Control intervention			
Lown (2017)	37	Healthy	29.35 ± 10.93	23.00 ± 2.27	4 (at least 2 days gap)	Reducose (capsule)	Maltodextrin, 50 g (250 ml)	a/0.125 g (6.75 mg) b/0.25 g (12.5 mg) c/0.5 g (25 mg)	Placebo^†^	Glucose (piAUC) Insulin (piAUC)	b, c b, c	Nausea Abdominal cramping Distension Flatulence
Wang (2018)	15	Healthy	23.80 ± 1.47	22.05 ± 1.56	7 (every 3 days)	Powder	4 kinds^∗∗^, 50 g (150 ml)	0.75 g (7.5 mg)	N.T	Glucose (iAUC) GI PPG	SMD 30 min SMD -	N.R
Kimura (2007)	24	Healthy	25.3 ± 0.7	20.9 ± 0.4	1	Powder	Sucrose, 50 g (100 ml)	a/0.4 g (6 mg) b/0.8 g (12 mg) c/1.2 g (18 mg)	Placebo (0 mg)	PPG PPI T-chol HDL TG HbA1C	b, c b, c − − − −	N.R
Asai (2011)	10	FBS 100–140	50.0 ± 10.6	24.3 ± 1.7	4 (every 2 weeks)	Capsule	Boiled white rice (200 g), dry seasoning (2 g)	a/3 mg b/6 mg c/9 mg	Placebo (0 mg)	PPG PPI	b, c 30 min a, b, c 30 min	Mild gas Bloating
Mudra (2007)	20	Healthy Diabetes	h/24–61 d/59–75	N.R	1	Extract	Sucrose, 75 g (500 ml)	1 g	Placebo^‡^	PPG GE Breath H_2_ test	First 120 min + +	N.R
Chung (2013)	50	FBS<125	22.7 ± 0.4	21.2 ± 0.4	1	Extract	Maltose, 75 g (50 ml)	a/1.25 g (4.5 mg) b/2.5 g (9 mg) c/5g (18 mg)	Placebo (0 mg)	Glucose (iAUC)	b, c 30, 60 min	N.R
Thaipitakwong (2019)	85	Healthy	a/25.81 ± 0.29 b/22.33 ± 5.57 d/23.31 ± 6.94 C/21.81 ± 5.72	a/20.57 ± 1.28 b/20.01 ± 1.47 d/20.51 ± 1.46 C/20.34 ± 1.41	1	Powder	Sucrose, 50 g (150 ml)	a/2.3 g (6 mg) b/4.6 g (12 mg) d/6.9 g (18 mg)	N.R	PPG Glucose (AUC)	a, b, d 30 min b, d 60 min d, +	Bloating Flatulence Loose stools

AUC, area under the curve; iAUC, incremental area under the curve; piAUC, positive incremental area under the curve; GI, glycemic index; SMD, sucrose, maltose, maltodextrin; PPG, postprandial glucose; PPI, postprandial insulin; T-chol, total cholesterol; HbA1C, hemoglobin A_1C_; FBS, fasting blood sugar; N.T, no treatment; N.R, not reported; TG, triglyceride; GE, glycemic excursion; C, control. ^*∗*^We use “+” if results were statistically significant and “–” if results were not statistically significant or have no difference. ^∗∗^Glucose, sucrose, maltose, maltodextrin. ^†^Microcrystalline cellulose 125 mg. ^‡^Red dye #40 and caramel.
